# Hydrogen Sulfide Attenuates Cisplatin-Induced Acute Kidney Injury via Dual Inhibition of Apoptosis and Pyroptosis

**DOI:** 10.3390/biomedicines13112696

**Published:** 2025-11-03

**Authors:** Zhenyuan Han, Yutao Jia, Dechao Yan, Ying Xue, Tianyu Deng, Ping Wang, Leijuan Xiao, Xiaoyan Wang

**Affiliations:** 1 Department of Nephrology, Nanjing BenQ Medical Center, The Affiliated BenQ Hospital of Nanjing Medical University, Nanjing 210019, China; h1220891885@163.com (Z.H.); jssqjyt@163.com (Y.J.);; 2 The Core Laboratory, Nanjing BenQ Medical Center, The Affiliated BenQ Hospital of Nanjing Medical University, Nanjing 210019, China

**Keywords:** hydrogen sulfide, cisplatin, acute kidney injury, pyroptosis, inflammation, apoptosis

## Abstract

**Purpose:** Cisplatin chemotherapy is complicated by acute kidney injury (cis-AKI), driven by regulated cell death pathways, including apoptosis and pyroptosis. However, the temporal relationship between apoptosis and pyroptosis in cis-AKI remains unclear. This study investigated the roles of these pathways and evaluated the renoprotective effect of the hydrogen sulfide (H_2_S) donor GYY4137. **Method:** Cis-AKI was modeled in mice and HK2 cells, divided into control, cisplatin, and cisplatin + GYY groups. Kidney function parameters, histopathology, and cell death were evaluated. Markers of apoptosis and pyroptosis, along with the H_2_S-producing enzyme, were analyzed. **Results:** Renal impairment progressed from BUN elevation to increased Scr, coupled with aggravated renal tissue damage. Apoptotic signaling peaked at 24 h, evidenced by a raised Bax/Bcl-2 ratio and caspase-3 cleavage. Pyroptosis pathways, via both NLRP3/caspase-1/GSDMD and caspase-3/GSDME axes, were activated later at 72 h, with concurrent rises in IL-1β and IL-18. GYY4137 treatment significantly ameliorated renal dysfunction, reducing serum creatinine and BUN levels by 22.64% and 22.5%, respectively. It suppressed both the early apoptotic and delayed pyroptosis cascades without reversing CBS downregulation. **Conclusions:** GYY4137 mitigated both apoptosis and pyroptosis, offering a promising multi-targeted therapy for cis-AKI.

## 1. Introduction

Acute kidney injury (AKI) occurs in nearly 35% of cisplatin-treated patients, which greatly limits its clinical use [1]. Tubular damage, inflammation, DNA damage, and oxidative stress are the typical features of renal toxicity of chemotherapy drugs, and tubular damage is the most serious. In the kidneys, chemotherapy drugs accumulate in tubular epithelial cells, causing cell death and AKI [2,3,4]. However, the molecular mechanism of cis-AKI has not been fully determined. Therefore, the early diagnosis and treatment of chemotherapeutic AKI need to be further studied.

In the pathophysiology of AKI, apoptosis and pyroptosis are two key death mechanisms; their occurrence and interaction have an important impact on the progression of kidney injury [5,6]. Cisplatin accumulates in renal proximal tubule epithelial cells after renal excretion and enters via membrane transporters, organic cation transporter 2 (OCT2) and copper transporter 1 (CTR1) [7,8]. It directly damages mitochondrial DNA and generates excessive reactive oxygen species (ROS), causing mitochondrial dysfunction and triggering apoptosis. Activated cysteine-aspartic protease 3 (caspase-3) mediates DNA repair failure, cytoskeleton degradation, and apoptotic body formation [9]. Elevated cleaved Caspase-3 levels in cisplatin-induced kidney injury models confirm apoptosis activation [9]. While pyroptosis has recently emerged as a critically involved inflammatory cell death process in the progression of AKI, its execution is predominantly mediated by members of the gasdermin protein family [10]. Upon activation, these proteins undergo proteolytic cleavage, releasing an N-terminal fragment that translocates to the plasma membrane. This fragment facilitates the formation of transmembrane pores, resulting in cell swelling, rupture, and the release of pro-inflammatory intracellular contents [6,11]. In AKI, a key trigger for pyroptosis is the assembly of the NOD-like receptor family pyrin domain-containing 3 (NLRP3) inflammasome, a multiprotein complex that responds to damage signals such as ROS and mitochondrial stress. The activated inflammasome promotes the cleavage of pro-cysteine-aspartic protease 1 (caspase-1) into its active form, which in turn proteolytically activates gasdermin D (GSDMD) to generate its pore-forming N-terminal domain (GSDMD-N), thereby initiating pyroptotic cell lysis [12]. Additionally, Caspase-3 can cleave GSDME to induce pyroptosis, particularly in renal tubular epithelial cells (RTECs) [13]. This dual activation of pyroptosis pathways exacerbates tubular injury and inflammation, contributing to the pathogenesis of AKI. Accumulating evidence further underscores the association of GSDME-mediated pyroptosis with various kidney pathologies, including AKI [14,15]. Apoptosis and pyroptosis are not mutually exclusive cell death modalities but may dynamically switch between each other [6]. Investigating their interactions and regulatory mechanisms may provide a foundation for developing targeted therapeutic strategies.

Hydrogen sulfide (H_2_S), the third endogenous signaling molecule following carbon monoxide and nitric oxide, holds a crucial physiological role in mammals. In the renal system, its biosynthesis is chiefly mediated by cystathionine-γ-lyase (CSE), cystathionine-β-synthase (CBS), and 3-mercaptopyruvate sulfurtransferase (3-MST) [16]. Approximately 75% of renal cells can express H_2_S synthetase, and all known pathways of H_2_S production have been identified in the kidneys; thus, H_2_S is closely involved in regulating renal physiological functions [17]. Cisplatin administration diminished H_2_S synthase expression, and the deficiency of endogenous H_2_S production further exacerbated the degree of kidney injury [18,19]. The supplementation of exogenous H_2_S reversed or alleviated the occurrence and development of various experimental acute kidney injuries [20]. To investigate the influence of H_2_S on apoptosis and pyroptosis in cis-AKI, we employed the slow-releasing donor GYY4137. Our findings are expected to provide new insights into the role of gasdermin family-mediated pyroptosis in chemotherapy-related nephrotoxicity.

## 2. Methods

### 2.1. Animal Model

Animal experiments were conducted under the supervision of the Committee on Animal Care at Nanjing Medical University, following the NIH Guidelines for Laboratory Animals (Approval No. IACUC-2312030, December 2023). Male *C57BL/6* mice, 7-week-old, weighing 17–20 g, were obtained from the experimental animal center of Jiangsu (Suzhou, China). Throughout the investigation period, mice maintained unrestricted access to standardized laboratory feed and water. Following a 7-day acclimatization period in controlled animal facilities maintaining temperatures between 18–25 °C with standardized 12 h light/dark cycles, these mice were randomly and equally divided into 3 three groups (*n* = 7/group): control group, cisplatin group, and cisplatin + GYY group. GYY4137 was administered intraperitoneally every 24 h for 4 injections (100 mg/kg). Before the second GYY4137 injection, cisplatin was delivered as a single intraperitoneal dose (20 mg/kg) [21]. In the control group, mice received 0.9% saline in place of GYY4137. On day 3 following cisplatin administration, animals were euthanized via cervical dislocation under anesthesia induced by 3% isoflurane, after which blood and kidney tissues were harvested for analysis. Additionally, a separate cohort of 18 mice was randomly allocated into three groups (*n* = 6/group): control group, 24 h cisplatin group, and 48 h cisplatin group. All operations were the same, and mice were euthanized 24/48 h post-injection.

### 2.2. Reagents

Abcam (Boston, MA, USA) antibodies: NLRP3 (rabbit monoclonal; 1:1000, ab263899), GSDMD (rabbit monoclonal; 1:2000, ab219800), Bcl-2(rabbit monoclonal, 1:1000, ab218123). Proteintech (Wuhan, China) antibodies: GSDME (rabbit polyclonal,1:1000, 13075-1-AP), caspase-3 (1:800, 19677-1-AP), Bax (rabbit polyclonal, 1:1000, 50599-2-Ig), caspase-1 (rabbit polyclonal, 1:1000, 31020-1-AP), CBS (rabbit polyclonal, 1:3000, 14787-1-AP), CSE(rabbit polyclonal, 1:2000, 12217-1-AP), GSDME (rabbit polyclonal,1:1000, 20770-1-AP). Cell Signaling Technology (Danvers, MA, USA) antibodies: IL-6 (rabbit monoclonal, 1:1500, 12912), MCP-1 (rabbit monoclonal, 1:1000, 41987). ABclonal Technology (Wuhan, China) antibodies: IL-18 (rabbit polyclonal, 1:1000, A20473), IL-1β (rabbit polyclonal, 1:1000, A22257,). Others: horseradish peroxidase (HRP)-conjugated Donkey Anti-Rabbit IgG (Ab205722, Abcam), HRP-conjugated Goat Anti-mouse IgG (bs-0296 G-HRP, Bioss, Beijing, China) or fluorophore-conjugated goat anti-mouse (D00115-05, LICOR, Beijing, China) or anti-rabbit IgG (D20125-35, LICOR), cisplatin (15663-27-1, Sigma-Aldrich, St. Louis, MO, USA), GYY4137 (HY-107632, Med-ChemExpress, Shanghai, China). RRIDs were seen in Supplementary Table S1.

### 2.3. Cell Culture

Renal epithelial cell lines below passage 20 were maintained in mycoplasma-free conditions using DMEM supplemented with 10% heat-inactivated FBS, antibiotic cocktail (100 U/mL penicillin and 100 μg/mL streptomycin), and incubated at 37 °C with 5% CO_2_ humidification. Following serum starvation for 1 h at 70–80% confluency in 6-well plates, experimental treatments were initiated using: (1) cisplatin (20 μM), (2) graded GYY4137 concentrations (50/100/200 μM), or (3) combination therapies—each supplemented with 5% FBS. Control groups received equivalent DMEM medium (M1902, Sigma-Aldrich). Post-48 h exposure, cellular suspensions were prepared for subsequent analytical procedures across eight experimental conditions: basal control, cisplatin monotherapy, three GYY4137 monodose groups, and three combination therapy cohorts.

### 2.4. Western Blot Analysis

Protein immunodetection was performed following established protocols [22]. Briefly, renal tissues or HK-2 lysates prepared with protease/phosphatase inhibitors (Beyotime Biotechnology, Shanghai, China) underwent 12% SDS-PAGE separation and nitrocellulose membrane transfer. After blocking with 5% skim milk/TBST, membranes received sequential incubations with primary antibodies (4 °C overnight) and species-matched HRP/fluorophore-conjugated secondary antibodies (1–2 h RT). Protein signals were detected using either chemiluminescence (BioRad ChemiDoc, Shanghai, China) or infrared imaging (LI-COR Odyssey, Lincoln, NE, USA), with quantitative normalization against β-actin/GAPDH performed via ImageJ ( Version 1.48V) analysis (NIH).

### 2.5. Histological Analysis

Renal histopathological analysis followed established methodologies [22,23,24]. Briefly, paraffin-embedded kidney sections underwent H&E and PAS staining after antigen retrieval. Tubular damage severity (graded 0–4) was determined by quantifying pathological features, 0, 1 ≤ 25%, 2 = 25–50%, 3 = 50–75%, and 4 ≥ 75%, including epithelial flattening, cast formation, and brush border loss across 10–20 randomly selected high-power fields (×800). It was conducted by two observers blinded to the treatment conditions. Parallel evaluation of apoptosis employed TUNEL assays on matched sections, with positive cells quantified per visual field (Carl Zeiss LSM 800 system). The apoptosis of tubular cells was detected using FITC-Labeled TUNEL Apoptosis Detection. Each mouse was selected for 10–20 randomly chosen fields (×800) to count TUNEL-positive cells/field. All microscopic images were observed using an LSM 800 confocal microscope (Carl Zeiss, Oberkochen, Germany).

### 2.6. I Immunofluorescence and Confocal Microscopy

Immunofluorescence staining was conducted per established methodologies [22,24,25]. Briefly, 2-µm kidney sections underwent dewaxing and ethanol-based rehydration. Following antigen retrieval and blocking procedures, sections received sequential incubations with: (1) primary antibodies against GSDME, GSDMD, caspase-3, and sodium–hydrogen exchanger 3 (NHE3; previously validated)/Aquaporin-1(AQP1, previously validated) at 4 °C overnight; (2) Alexa Fluor-conjugated secondary antibodies (Invitrogen, Shanghai, China) with DAPI counterstaining under dark conditions. Processed specimens from two mice per group were mounted in antifade medium (Citotest Scientific, Nanjing, China) and imaged using a Zeiss LSM 800 system.

### 2.7. H_2_S Determination

The quantification of H_2_S was performed using a colorimetric method based on the reaction of H_2_S with N, N-dimethyl-p-phenylenediamine and ferric ammonium sulfate to form methylene blue. Tissue samples were homogenized in extraction solution at a ratio of 1:5–10, followed by centrifugation to collect the supernatant. For cellular samples, cells were collected and sonicated in extraction solution at a ratio of 1:1, with subsequent centrifugation to obtain the supernatant. The absorbance of the reaction mixture was measured at 680 nm, and the content was calculated according to the formula provided by the manufacturer. The H_2_S assay kit was purchased from Beijing Solarbio Science & Technology Co., Ltd. (Beijing, China).

### 2.8. JC-1 Measurement

Consistent with established mitochondrial physiology protocols [26], JC-1 assay implementation followed standardized membrane potential evaluation principles. HK-2 cells were seeded in 6-well plates under standard culture conditions. JC-1 reagent was reconstituted per manufacturer specifications prior to 20 min substrate loading (M34152, Thermo Fisher Scientific, Waltham, MA, USA). Post-staining procedures included sequential media aspiration, two-cycle buffer washing, and final resuspension in 500 μL fresh buffer for flow cytometric quantification.

### 2.9. Transmission Electron Microscope (TEM)

Fresh tissue samples (1 mm^3^) were immediately immersed in primary EM fixative for initial immobilization, followed by transfer to fresh fixative solution in microcentrifuge tubes at 4 °C. After phosphate buffer rinsing (0.1 M, pH 7.4), post-fixation was performed with 1% osmium tetroxide for 2 h. Following the ethanol dehydration series, samples underwent acetone-epoxy resin gradient infiltration before final embedding in pure EPON812 resin. Ultrathin sections (60–80 nm thickness) were prepared using an ultramicrotome, stained with uranyl acetate-lead citrate, and imaged under Hitachi transmission electron microscopes (HT7800/HT7700).

### 2.10. Quantitative Real-Time PCR

RNA analysis followed standardized molecular biology protocols. Total RNA isolated with Trizol^®^ reagent underwent reverse transcription using ReverTra Ace (Vazyme, Nanjing, China). Quantitative PCR amplifications (40 cycles) were performed on an Applied Biosystems 7300 system (Thermo Fisher Scientific, MA, USA) with Vazyme chemistry, employing β-actin-normalized 2^−ΔΔCT^ analysis. Primer sequences are detailed in Supplementary Table S2. Experimental methodology was implemented according to previously published techniques in our laboratory [22,24].

### 2.11. Cell Viability/Toxicity Assays

Cell proliferation was assessed with a CCK-8 assay system (Enogene Biotech Co., Ltd., Nanjing, China). Optical density measurements at 450 nm were obtained using a microplate reader (SpectraMax iD3, Molecular Devices, Shanghai, China) for quantitative analysis. Results were normalized to untreated controls and expressed as relative viability percentages. Cytotoxic effects were evaluated through lactate dehydrogenase (LDH) leakage quantification using commercial detection reagents (Beyotime Biotechnology, Nantong, China), with enzymatic activity measurements performed according to the supplier’s specifications. The cytotoxicity index was calculated as the percentage ratio of extracellular LDH activity to total cellular LDH content.

### 2.12. Statistical Analysis

All data generated in this study were included in the reported analyses. Statistical analyses were performed using GraphPad Prism (Version 8.0, GraphPad Software, San Diego, CA, USA) and SPSS Statistics (Version 21.0, IBM Corp., Armonk, NY, USA). The data are presented as the mean ± standard error of the mean (SEM). Prior to comparative analysis, the normality of data distribution was verified using the D’Agostino-Pearson omnibus and Shapiro-Wilk tests. No significant outliers were identified and excluded from the datasets. For comparisons among multiple groups, one-way analysis of variance (ANOVA) was employed, followed by the Holm–Šídák post hoc test for further pairwise analysis. A *p*-value of less than 0.05 was considered statistically significant.

## 3. Results

### 3.1. Impact of GYY4137 on Renal Function in cis-AKI Mice

**Figure 1:**
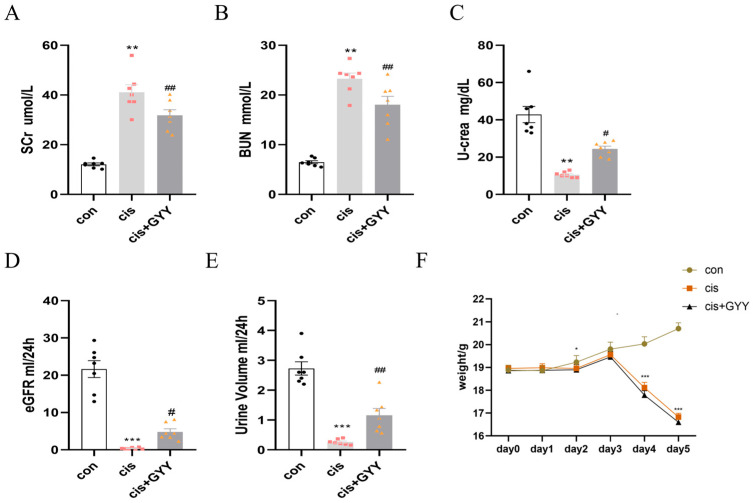
Therapeutic effects of exogenous H_2_S on renal function. (**A**,**B**) Scr and BUN levels; (**C**) Urinary creatinine excretion; (**D**) estimated glomerular filtration rate; (**E**) urine output measurement; (**F**) body weight changes. Data represent the mean ± SEM (n = 7/group). Statistical significance: * *p* < 0.05, ** *p* < 0.01, *** *p* < 0.001 (control vs. cisplatin); # *p* < 0.05, ## *p* < 0.01, ### *p* < 0.001 (cisplatin vs. cisplatin + GYY).

The AKI mouse model was produced by intraperitoneal cisplatin injection in *C57BL/6* mice. We did not impose any restrictions on food and water. Physiological assessments following 72 h cisplatin exposure revealed altered renal parameters. Biological sampling demonstrated elevated serum creatinine (Scr) and blood urea nitrogen (BUN) levels concurrent with reduced urinary creatinine excretion (Figure 1A–C). Estimate glomerular filtration rate (eGFR), calculated according to SCr, urine creatinine, and urine volume decreased significantly in the cisplatin group (Figure 1D,E) [27]. With the injection of GYY4137, these changes were reversed. Mice exhibited a reduction in body weight starting on day 3, with further decline on day 5, and GYY4137 treatment failed to mitigate cisplatin-induced weight loss (Figure 1F).

### 3.2. Impact of GYY4137 on H_2_S-Producing Enzymes in cis-AKI Mice

**Figure 2:**
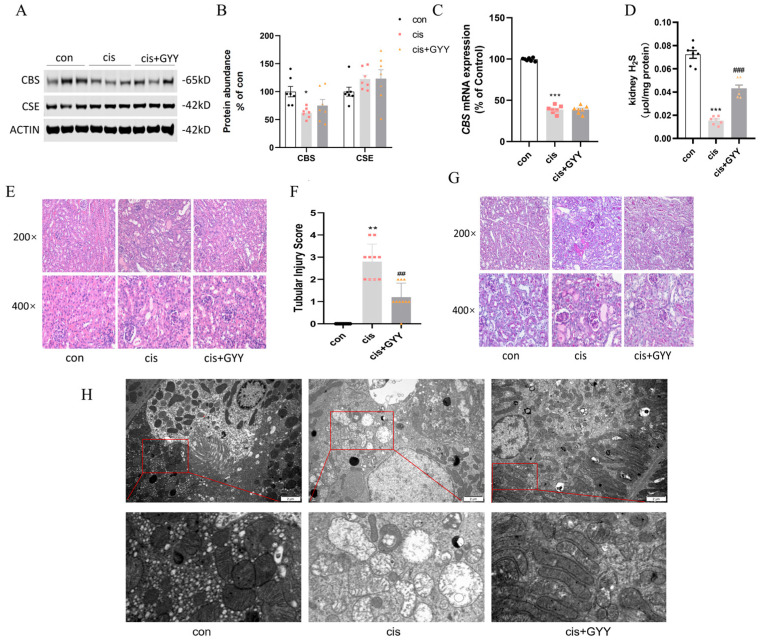
Renal CBS was reduced in cis-AKI mice, and H_2_S alleviated the pathological damage. (**A**) Representative Western blot images of CBS and CSE expression. (**B**) Quantitative analysis of enzyme protein levels. (**C**) mRNA expression of CBS. (**D**) Kidney H_2_S levels. (**E**) H&E staining showing characteristic tubular damage. (**F**) Quantitative assessment of pathological injury scores. (**G**) PAS-stained renal sections. The cisplatin-treated mice exhibited impairment in AKI pathological changes, such as vacuolar degeneration of tubular epithelium, brush border loss, and structural disorganization (200× and 400× magnification; scale bars = 50 μm). The GYY-treated mice alleviated renal tubular injury. (**H**) Mitochondria in a transmission electron microscope (scale bars = 2 μm, second row: 500 nm). Data represent the mean ± SEM (n = 7/group). Statistical significance: * *p* < 0.05, ** *p* < 0.01, *** *p* < 0.001 (control vs. cisplatin); # *p* < 0.05, ## *p* < 0.01, ### *p* < 0.001 (cisplatin vs. cisplatin + GYY).

We quantified renal cortical expression of key H_2_S synthase enzymes (CSE and CBS) following cisplatin exposure. CBS expression was significantly reduced in AKI mice (Figure 2A,B), while CSE levels remained unaffected. This suppression pattern was conserved at both transcriptional and translational levels for CBS (Figure 2C). After exogenous administration of H_2_S, the CBS did not recover at both the protein and mRNA levels. Kidney H_2_S levels decreased after cisplatin, and exogenous supplementation partially restored the content (Figure 2D).

### 3.3. Impact of GYY4137 on Renal Pathological Damage in cis-AKI Mice

Based on H&E and PAS staining, mice with cisplatin exhibited significant pathological alterations, such as vacuole-like degeneration of renal tubular epithelial cells, absence of brush borders, and a disrupted cell tubule pattern. Under TEM, kidney tissues from the cisplatin group exhibited mitochondrial enlargement accompanied by vacuolization, matrix density reduction, and cristae disruption or loss. After GYY treatment, these pathological changes were reversed, demonstrating a significantly reduced tubular injury score (Figure 2E–H).

### 3.4. Impact of GYY4137 on Apoptosis, Pyroptosis, and Inflammation in cis-AKI

**Figure 3:**
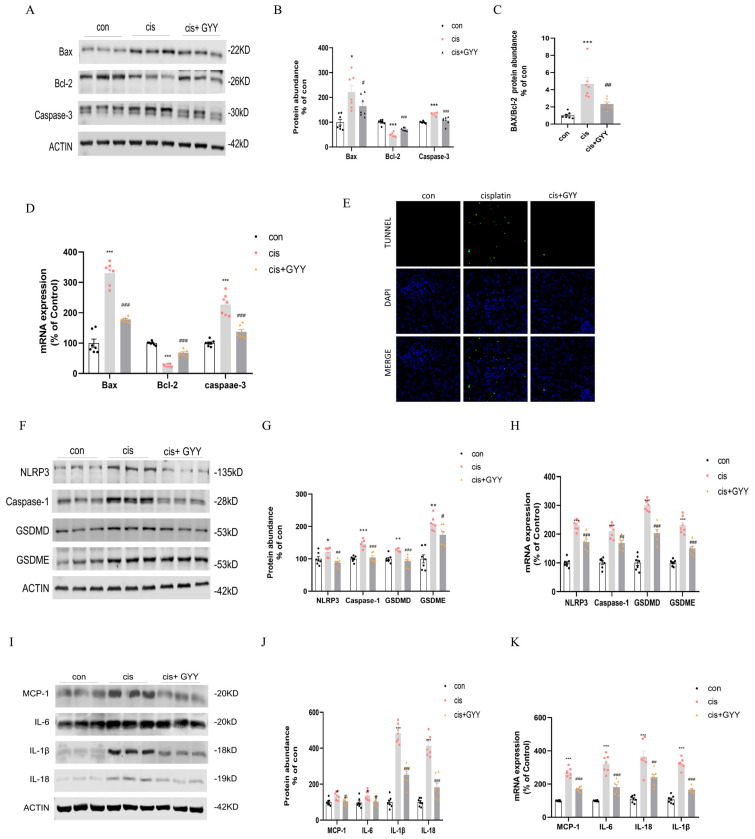
H_2_S attenuated cisplatin-induced renal proximal tubular cell death and inflammatory responses. (**A**) Western blot analysis of Bax, Bcl-2, and Caspase-3 protein expression. (**B**) Quantification of Bax, Bcl-2, and Caspase-3 protein levels. (**C**) Bax/Bcl-2 ratio comparison. (**D**) mRNA expression of Bax, Bcl-2, and Caspase-3. (**E**) TUNEL/DAPI co-staining illustrating apoptotic nuclei (green) and total nuclei (blue; scale bar = 50 µm). (**F**) Western blot detection of NLRP3, Caspase-1, GSDMD, and GSDME. (**G**) Protein quantification of NLRP3, Caspase-1, GSDMD, and GSDME. (**H**) mRNA levels of NLRP3, Caspase-1, GSDMD, and GSDME. (**I**) Western blot of inflammatory cytokines MCP-1, IL-6, IL-1β, and IL-18. (**J**) Protein level quantification of inflammatory cytokines. (**K**) mRNA expression of inflammatory markers. Data represent the mean ± SEM (n = 7/group). Statistical significance: * *p* < 0.05, ** *p* < 0.01, *** *p* < 0.001 (control vs. cisplatin); # *p* < 0.05, ## *p* < 0.01, ### *p* < 0.001 (cisplatin vs. cisplatin + GYY).

We observed dysregulation of apoptosis-related proteins in renal cortical tissue, characterized by upregulated Bax and caspase-3 expression, concurrent with Bcl-2 suppression. These changes were reversed after exogenous administration of GYY4137 (Figure 3A–C). qPCR analysis revealed that alterations in Bax, Bcl-2, and caspase-3 mRNA levels exhibited concordant variation with their corresponding protein expression (Figure 3D). TUNEL experiments showed that cisplatin activated tubular cell apoptosis, while GYY4137 intervention reduced the percentage of positive renal tubular cells (Figure 3E). The necrotic biomarkers NLRP3, caspase-1, GSDMD, and GSDME were upregulated, and H_2_S dampened these changes (Figure 3F,G). qPCR demonstrated consistency with their protein level (Figure 3H). Inflammatory factors were released during pyroptosis, Western blot analysis and qPCR showed that IL-6, IL-18, IL-1β, and MCP-1 were substantially upregulated by cisplatin and suppressed by co-administration of GYY4137 (Figure 3I–K).

**Figure 4:**
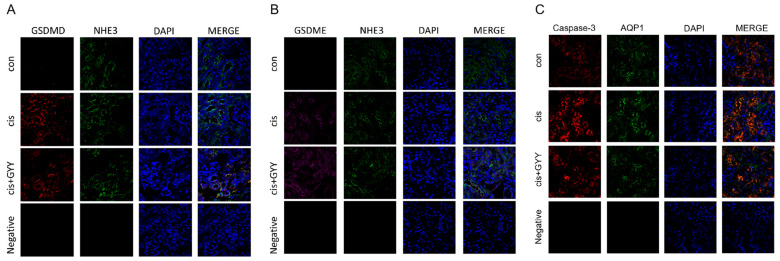
Modulation of gasdermin proteins by GYY4137 in cisplatin-induced kidney injury. (**A**) Cortical tubular co-localization of GSDMD (red, rabbit antibody) with NHE3 (green, chicken antibody) visualized by confocal microscopy (400×). (**B**) Detection of GSDME (purple) and NHE3 (green) in proximal tubule brush borders. (**C**) Detection of Caspase-3 (red) and AQP1 (green) in proximal tubule brush borders. Nuclear counterstaining: DAPI (blue). n = 3. Scale bars = 50 μm.

To investigate the spatial expression of gasdermin proteins in renal tissues, dual immunofluorescence staining was performed for GSDMD/NHE3 and GSDME/NHE3, utilizing NHE3 as a definitive apical membrane marker of proximal tubules [24]. Fluorescence microscopy revealed distinct co-localization signals (manifested as yellow regions in merged channels) along the brush border membranes, confirming the tubular epithelial localization of both gasdermin proteins. GSDMD and GSDME immunoreactivity was weaker in cisplatin + GYY4137-treated specimens compared with cisplatin-exposed controls (Figure 4A,B). These observations corroborated that GYY4137 inhibited cisplatin-induced apoptosis, pyroptosis, and inflammation. Additionally, co-localization in renal proximal tubules demonstrated concurrent expression of caspase-3 with AQP1, an established apical membrane marker [25], exhibiting distribution patterns concordant with GSDME/GSDMD expression trends (Figure 4C).

### 3.5. Concentration-Dependent Cytotoxicity of Cisplatin and Dose Optimization of GYY4137 in HK-2 Cells

**Figure 5:**
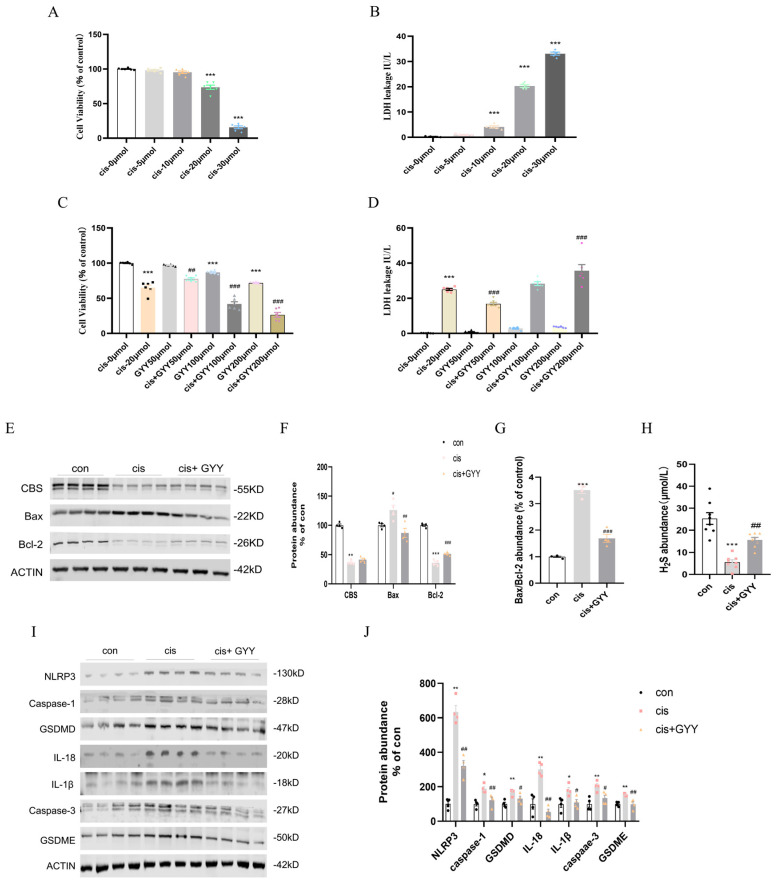
Cytotoxicity of cisplatin, dose optimization of GYY4137, and the effect of H_2_S on HK-2 cells. (**A**) Dose-dependent reduction in HK-2 cellular survival with cisplatin exposure (0, 5, 10, 20, 30 μmol/L; n = 6). (**B**) Cisplatin-induced cytotoxicity quantified through LDH leakage (n = 5). (**C**) Combinatorial assessment of GYY4137 (0–200 μmol/L) therapeutic efficacy: Baseline viability (CCK-8 assay) and cisplatin (20 μmol/L)-challenged cytoprotection (n = 6). (**D**) The effect of different concentrations of GYY4137, ranging from 0 to 200 μmol/L, with or without cisplatin (20 μmol/L) exposure on cellular toxicity as tested using the LDH assay (n = 6). (**E**) Representative Western blot of Bax, Bcl-2, and CBS (n = 4). (**F**) Quantitative data on Bax, Bcl-2, and Caspase-3 protein levels. (**G**) The protein level of Bax/Bcl-2. (**H**) H_2_S abundance in cells. (**I**) Representative Western blot of NLRP3, Caspase-1, GSDMD, IL-18, IL-1β, Caspase-3, and GSDME (n = 4). (**J**) Quantitative data on NLRP3, Caspase-1, GSDMD, IL-18, IL-1β, Caspase-3, and GSDME protein levels. Data represent the mean ± SEM (n = 4/group). Statistical significance: * *p* < 0.05, ** *p* < 0.01, *** *p* < 0.001 (control vs. cisplatin); # *p* < 0.05, ## *p* < 0.01, ### *p* < 0.001 (cisplatin vs. cisplatin + GYY).

At 20 μM cisplatin, cell viability significantly decreased compared to the control, while 30 μM cisplatin caused near-complete cell death (Figure 5A). LDH release mirrored this trend, confirming cisplatin cytotoxic effects (Figure 5B). Protein analysis revealed CBS downregulation and GSDME/GSDMD activation at 20 μM cisplatin (Supplementary Figure S1). To evaluate GYY4137’s therapeutic potential, HK-2 cells were exposed to 20 μM cisplatin in combination with varying GYY4137 concentrations (50, 100, 200 μM). CCK-8 and LDH release assays demonstrated that 50 μM GYY4137 significantly attenuated cisplatin-induced cytotoxicity, whereas higher concentrations (100–200 μM) failed to protect against damage (Figure 5C,D). Results confirmed that 50 μM GYY4137 suppressed cisplatin-triggered GSDME and GSDMD cleavage, while higher doses showed no significant effects (Supplementary Figure S2).

### 3.6. GYY4137 Modulates Cisplatin-Induced Cell Death in HK2 Cells

20 μM cisplatin and 50 μM GYY4137 were selected for subsequent experiments. After 48 h of treatment, it protected HK2 cells from cisplatin-induced injury. CBS, Bcl-2 downregulation, and Bax upregulation were observed. And GYY4137 mitigated these changes without restoring endogenous CBS expression (Figure 5E–G). Consistent with in vivo observations, cisplatin-exposed cells diminished H_2_S levels, which were recoverable through exogenous supplementation (Figure 5H). Cisplatin exposure up-regulated pyroptosis-related pathways (NLRP3/caspase-1/GSDMD, and caspase-3/GSDME) and inflammatory factors. H_2_S treatment showed down-regulated pyroptosis activation and mitigated the production of IL-18 and IL-1β (Figure 5I,J). Therefore, H_2_S supplementation could reduce apoptosis and pyroptosis in vitro. Mitochondrial membrane potential was increased in HK-2 cells treated with GYY4137 compared with cisplatin(Supplementary Figure S3). The results were validated in mouse proximal convoluted tubule cells (mPTCs) (Supplementary Figure S4).

### 3.7. Time-Course Analysis of Renal Function and Pathology in cis-AKI

**Figure 6:**
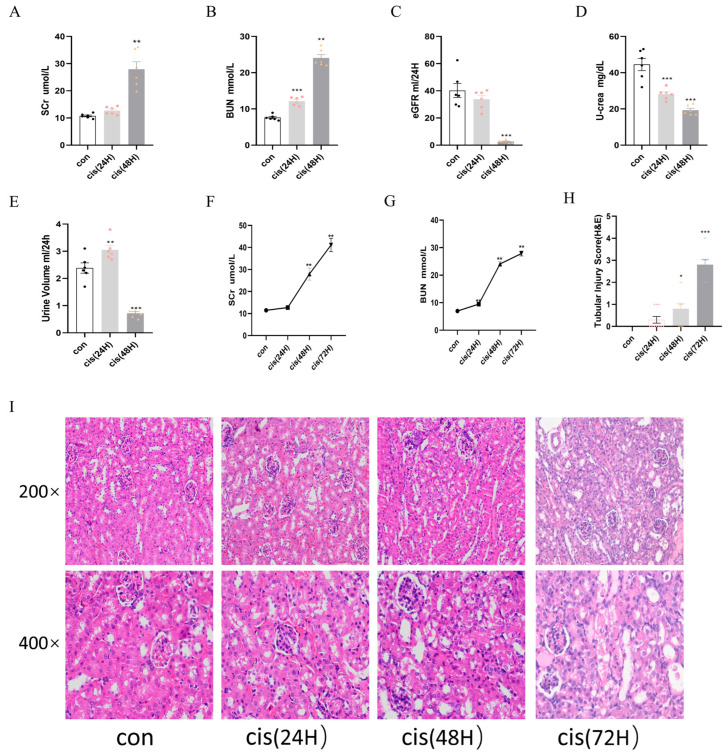
Time-course analysis of renal function and pathology in cis-AKI progression. Mice were allocated to three experimental cohorts (n = 6/group): untreated controls, 24 h cisplatin-treated, and 48 h cisplatin-treated. (**A**,**B**) Scr and BUN levels. (**C**) Estimated glomerular filtration rate. (**D**) Urinary creatinine excretion. (**E**) Diuresis volume measurement. (**F**,**G**) Temporal progression of Scr and BUN. (**H**,**I**) Histopathological evaluation through H&E staining with corresponding semi-quantitative tubular damage scoring. Data represent the mean ± SEM (n = 6/group). Statistical significance: * *p* < 0.05, ** *p* < 0.01, *** *p* < 0.001 (control vs. cisplatin).

Consistent with cisplatin-induced nephrotoxicity, serum biomarkers of renal function exhibited time-dependent alterations. BUN levels increased at 24 h, while Scr rose markedly at 48 h, coinciding with a significant decline in eGFR (Figure 6A–C). Urinary creatinine excretion decreased at 24 h, followed by oliguria at 48 h (Figure 6D,E). BUN and Scr levels changed from baseline and peaking on day 3 (Figure 6F,G). Histopathological analysis revealed that granular tubular patterns, exfoliation and necrosis of luminal cells, and the absence of brush edges were observed in tubular epithelial cells over time (Figure 6H,I).

### 3.8. Time-Course Analysis of H_2_S-Producing Enzymes, Apoptosis, and Pyroptosis in cis-AKI

**Figure 7:**
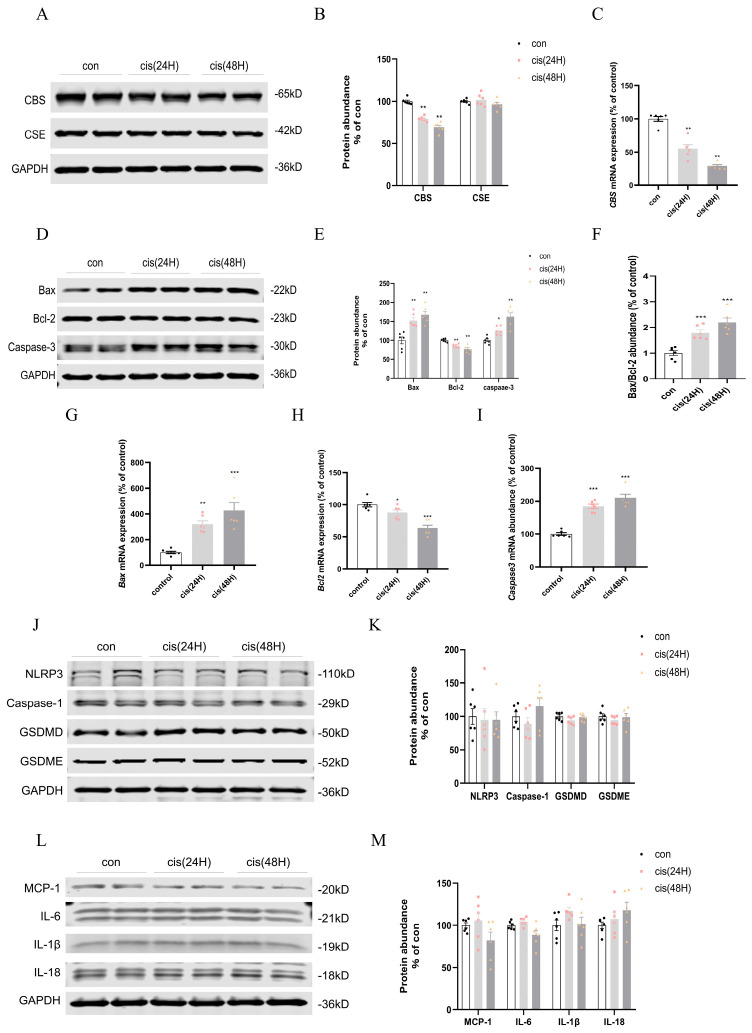
Temporal modulation of H_2_S-synthesizing enzymes and cell death pathways in cisplatin nephrotoxicity. (**A**) CBS/CSE Western blot. (**B**) CBS/CSE protein quantification. (**C**) mRNA level of CBS. (**D**) Bax/Bcl-2/Caspase-3 Western blot. (**E**) Bax/Bcl-2/Caspase-3 protein quantification. (**F**) Bax/Bcl-2 dynamics. (**G**–**I**) Bax/Bcl-2/Caspase-3 mRNA expression. (**J**) NLRP3/Caspase-1/GSDMD/GSDME Western blot. (**K**) NLRP3/Caspase-1/GSDMD/GSDME mRNA expression. (**L**) MCP-1/IL-6/IL-18/IL-1β Western blot. (**M**) MCP-1/IL-6/IL-18/IL-1β mRNA expression. Data represent mean ± SEM (n = 6/group). Statistical significance: * *p* < 0.05, ** *p* < 0.01, *** *p* < 0.001 (control vs. cisplatin).

The early suppression of CBS (24 h) promoted apoptotic activation (Figure 7A–C), implicating H_2_S deficiency in mitochondrial dysfunction. In the 24 h cisplatin group, alterations in apoptotic regulators were observed. Bax upregulated while anti-apoptotic Bcl-2 levels suppressed, accompanied by elevated Caspase-3 (Figure 7D–F). Changes were shown at the mRNA level (Figure 7G–I). These findings indicated early activation of mitochondrial apoptosis within 24 h of cisplatin exposure. In contrast, pyroptosis-related markers—NLRP3, Caspase-1, GSDMD, IL-1β, IL-18, IL-6, MCP-1, and GSDME—remained unchanged at both 24 and 48 h, suggesting delayed involvement of pyroptosis in cisplatin nephrotoxicity (Figure 7J–M). In vitro, analysis demonstrated absent pyroptosis activation in HK2 cells exposed to cisplatin for both 12/24 h durations (Supplementary Figure S5).

## 4. Discussion

Although cis-AKI is not rare in oncological patients clinically, there are limited prevention and treatment methods available, except for adequate hydration [28]. Basic researchers have explored H_2_S nephroprotective efficacy against cisplatin-associated nephrotoxicity, with demonstrated suppression of inflammation, ROS production, apoptosis, tubule cell death, and renal impairment [18,20,29]. In this study, we revealed that cisplatin triggered a coordinated activation of mitochondrial apoptosis and NLRP3 inflammasome-driven pyroptosis, culminating in epithelial demise, inflammation, and functional filtration decline. Comparative pharmacodynamic evaluation revealed GYY4137 therapeutic superiority over conventional H_2_S donors (e.g., NaHS, Na_2_S_4_), attributable to its enhanced controlled-release profile with sustained bioactivity modulation [21,29,30]. By utilizing both in vivo and in vitro cis-AKI models, GYY4137 reversed these changes, underlining its role as a potential candidate for kidney protection for cis-AKI.

Cisplatin positively charged metabolites accumulate in mitochondria, making mitochondrial DNA more vulnerable than nuclear DNA [31]. Cellular sensitivity correlates with mitochondrial density and membrane potential, explaining why renal proximal tubules—rich in mitochondria—are primary cisplatin toxicity targets [32,33]. Cisplatin induced Bcl-2 family proteostasis collapse through reciprocal Bax/Bcl-2 expression switching, initiating a mitochondrial apoptosis cascade characterized by membrane permeabilization kinetics, subsequent cytochrome C mobilization into the cytosol, and terminal caspase-3 effector activation [1,34,35]. Caspase-3, in turn, executed apoptosis by cleaving substrates like PARP, resulting in DNA fragmentation and apoptotic body formation [36]. GYY4137 counteracted this cascade by upregulating Bcl-2 expression and inhibiting Bax mitochondrial translocation, stabilizing mitochondrial integrity.

Irrespective of its underlying cause, inflammation is characteristic and common in AKI pathogenesis, and emerging studies indicate that pyroptosis could be a critical factor [15,37]. Previous research found that suppression of the NLRP3 axis-regulated pyroptosis mitigated diverse forms of AKI. For instance, Li et al. identified that macrophage migration inhibitory factor exacerbates sepsis-related renal damage through activation of the NF-κB/NLRP3 signaling cascade [38]. Similarly, inhibition of thrombospondin1 was shown to attenuate sepsis-induced AKI by modulating NLRP3 inflammasome activity [39]. Furthermore, NLRP3 inflammasome activation, accompanied by pyroptosis and inflammatory responses, has been documented in ischemia/reperfusion and cisplatin-induced injury models [40,41]. These findings collectively highlighted the involvement of the NLRP3-mediated pyroptosis pathway in multiple AKI etiologies. In our study, upregulation of NLRP3-aix was observed, underscoring the pathogenic role of NLRP3-dependent pyroptosis in cisplatin-associated AKI. Notably, subsequent studies revealed that beyond inflammatory caspases, pyroptosis could also be triggered by apoptosis-associated caspases. Chemotherapy agents activated caspase-3, subsequently cutting GSDME, releasing its N-terminal, and causing cell lysis from apoptosis to pyroptosis [13]. While caspase-1-dependent pyroptosis has been well described [42], new evidence links caspase-3-mediated GSDME cleavage to tubular cell death via membrane pore formation in cis-AKI. GSDME deficiency markedly inhibited cell death in AKI models, concurrently alleviating tubular damage and inflammatory responses [15]. These findings align with our experimental observation, which demonstrated a marked upregulation of caspase-3 in both renal tissues and HK2 cells following cisplatin treatment, concurrent with an elevation in GSDME levels. With the use of GYY4137, we observed pharmacological inhibition of caspase-3, leading to subsequent deactivation of GSDME. By blocking caspase-3, the rupture of renal tubular epithelial cells (TECs) and the release of inflammatory cytokines were reduced, thereby mitigating renal functional decline.

To describe the progression of cisplatin-induced renal injury, we added measurements of renal function, apoptosis, and pyroptosis 24 and 48 h after cisplatin administration. Bax/Bcl-2 imbalance indicated early activation of mitochondrial apoptosis within 24 h of cisplatin exposure. However, pyroptosis-related markers were still unchanged, suggesting delayed involvement of pyroptosis in cisplatin nephrotoxicity. At 72 h, pyroptosis was activated, demonstrating a temporal shift in cell death mechanisms, with apoptosis dominating early injury and pyroptosis contributing to later-stage damage. Additionally, the staggered rise in BUN and Scr aligned with the sequential activation of apoptosis and pyroptosis, suggesting that early tubular apoptosis initiated subclinical injury, detectable via BUN, while later pyroptosis exacerbated functional decline, reflected in creatinine elevation.

The temporal correlation between CBS loss and apoptosis initiation implied that endogenous H_2_S depletion may sensitize renal tubular cells to cisplatin-induced mitochondrial apoptosis. In GYY4137-treated mice, kidney H_2_S abundance partially recovered, and CBS expression did not recover, but GYY4137 supplementation inhibited apoptosis/pyroptosis, suggesting that its protective effects were mediated through exogenous H_2_S delivery. Methylene blue-based assays indicate plasma H_2_S concentrations generally range between 10–100 μM, though the precise determination of its physiological steady-state levels remains methodologically contentious [16,43]. H_2_S homeostasis involves tightly regulated biosynthesis and catabolism pathways. Super physio-logical H_2_S concentrations selectively impair mitochondrial complex IV activity, reducing oxygen consumption and ATP generation [44]. In vitro, concentration-dependent cytotoxicity of GYY4137 at doses exceeding 100 μM, as evidenced by increased lactate dehydrogenase (LDH) release. Mechanistically, physiological H_2_S enhances mitochondrial preservation through (1) cAMP/PKA axis-mediated potentiation of complex II-driven electron transport chain activity; (2) direct ATP synthase activation via persulfidation modification; and (3) dynamic regulation of mitochondrial fusion/fission equilibrium [45,46,47]. In cisplatin-treated HK-2 cells, we observed marked mitochondrial membrane depolarization, which GYY4137 supplementation reversed through transmembrane potential restoration.

**Figure 8:**
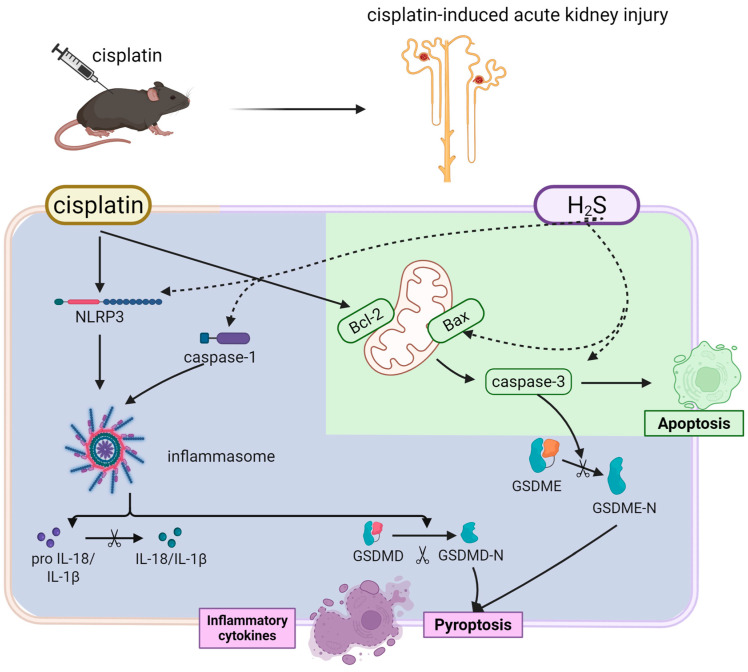
H_2_S attenuates cisplatin-induced acute kidney injury via dual suppression of apoptosis (green) and pyroptosis (purple). Cisplatin triggers biphasic renal tubular injury (solid line). The H_2_S donor GYY4137 protects against these effects by (dashed line).

Combined with our previous data, these results delineated that early apoptosis dominated, driven by mitochondrial dysfunction and CBS downregulation, leading to subclinical tubular injury. Pyroptosis emerged via NLRP3 inflammasome activation and GSDMD/GSDME cleavage, exacerbating inflammation and overt renal failure. Thus, cis-AKI followed a time-resolved pathological cascade: early CBS suppression and mitochondrial apoptosis initiated tubular injury, followed by NLRP3 inflammasome-driven pyroptosis that amplified inflammation and renal dysfunction. This temporal pattern underscored apoptosis as the primary driver of initial injury, while pyroptosis contributed to progressive damage and clinical symptom onset (Figure 8). These findings highlighted the therapeutic potential of H_2_S donors in mitigating cisplatin-induced renal injury, even in impaired endogenous H_2_S synthesis. Beyond our findings, H_2_S may indirectly influence regulated cell death (RCD) pathways through shared mechanisms. Mitochondrial dysfunction and ROS overproduction constitute shared pathological features in ferroptosis, pyroptosis, and necroptosis. The preservation of mitochondrial integrity and stabilization of redox homeostasis by H_2_S could potentially suppress both inflammasome-mediated pyroptosis and RIPK3/MLKL-dependent necroptosis [29]. Future studies systematically examining the cross-talk between H_2_S signaling and these RCD pathways under cisplatin-induced stress conditions could elucidate critical determinants of renal tubular cell survival and inform combination therapies simultaneously targeting multiple RCD mechanisms in acute kidney injury.

Overall, this study elucidated the temporal dynamics and mechanistic interplay between apoptosis and pyroptosis in cis-AKI while highlighting the therapeutic potential of the H_2_S donor GYY4137. These findings underscore the therapeutic advantage of multi-modal interventions in cis-AKI, where single-pathway approaches often falter due to compensatory mechanisms. AP39, a novel mitochondria-targeted hydrogen sulfide (H_2_S) donor, demonstrates renoprotective effects through coordinated antioxidant, anti-inflammatory, and anti-apoptotic mechanisms. Unlike conventional H_2_S donors, its triphenylphosphonium-mediated mitochondrial delivery system achieves organelle-specific H_2_S enrichment, simultaneously enhancing tricarboxylic acid cycle efficiency for ATP homeostasis and reducing systemic toxicity [48,49,50]. Current preclinical evidence confirms favorable dose tolerance across in vitro and rodent models. Future investigations should focus on developing renal-selective delivery platforms, establishing cross-species therapeutic validation frameworks, and conducting longitudinal safety assessments to bridge the pre-clinical-clinical translation gap.

## 5. Limitation

This study has certain limitations. Firstly, the lack of interventional approaches using specific inhibitors for H_2_S enzymes, apoptosis or pyroptosis prevents a definitive conclusion on which cell death pathway predominates in driving cisplatin-induced kidney injury. Our subsequent research will employ these inhibitors to dissect their individual contributions. Secondly, while GYY4137 demonstrated concurrent suppression of both pathways, the deeper molecular mechanisms underlying H_2_S action remain incompletely elucidated. We plan to investigate the specific upstream signaling and protein targets of H_2_S to provide more direct mechanistic insights.

## Data Availability

The data that support the findings of this study are available in the manuscript. Extra data used to support this study are available from the corresponding author upon request.

## Supplementary Materials

The following supporting information can be downloaded at https://www.mdpi.com/article/10.3390/biomedicines13112696/s1. Figure S1. Concentration-dependent cytotoxicity of cisplatin and pyroptosis proteins in HK-2 cells. Figure S2. Concentration optimization of GYY4137 in HK-2 cells. Figure S3. Mitochondrial membrane potential of HK-2 cells. Figure S4. GYY4137 modulates cisplatin-induced cell death in mPTCs. Figure S5. Time-course analysis of H2S-producing enzymes and pyroptosis in HK-2 cells. Table S1. RRIDs of antibodies. Table S2. The primers used for qRT-PCR are listed below.

## Abbreviations

The following abbreviations are used in this manuscript:

CBS	cystathionine-beta-synthase
CSE	cystathionine-gamma-lyase
3-MST	3-mercaptopyruvate sulfurtransferase
AKI	acute kidney injury
CIS	cisplatin
H_2_S	hydrogen sulfide
BAX	Bcl-2-associated X protein
BCL-2	B-cell lymphoma 2 protein
Caspase-3	cysteine-aspartic protease 3
NLRP3	NOD-like receptor family pyrin domain containing 3
Caspase-1	cysteine-aspartic protease 1
IL-18	interleukin-18
IL-1β	interleukin-1 beta
HK2	human renal tubular epithelial cells
NHE3	sodium–hydrogen exchanger isoform 3
AQP1	aquaporin-1
IL-6	interleukin-6
MCP-1	monocyte chemoattractant protein-1
OCT2	organic cation transporter 2
CTR1	copper transporter 1
ROS	reactive oxygen species
TEM	transmission electron microscope
SCR	serum creatinine
BUN	blood urea nitrogen
eGFR	estimated glomerular filtration rate
CCK-8	cell counting kit-8
LDH	lactate dehydrogenase
RCD	regulated cell death
GSDMD/E	gasdermin D/E
