# Supplementary material for: Hydrogen Sulfide Attenuates Cisplatin-Induced Acute Kidney Injury via Dual Inhibition of Apoptosis and Pyroptosis
Source: Biomedicines. 2025 Nov 3;13(11):2696. doi: 10.3390/biomedicines13112696 (PMC12649982; doi:10.3390/biomedicines13112696)
Supplement: Supplementary file 1 [file biomedicines-13-02696-s001.zip › biomedicines-3880604-supplementary.pdf]

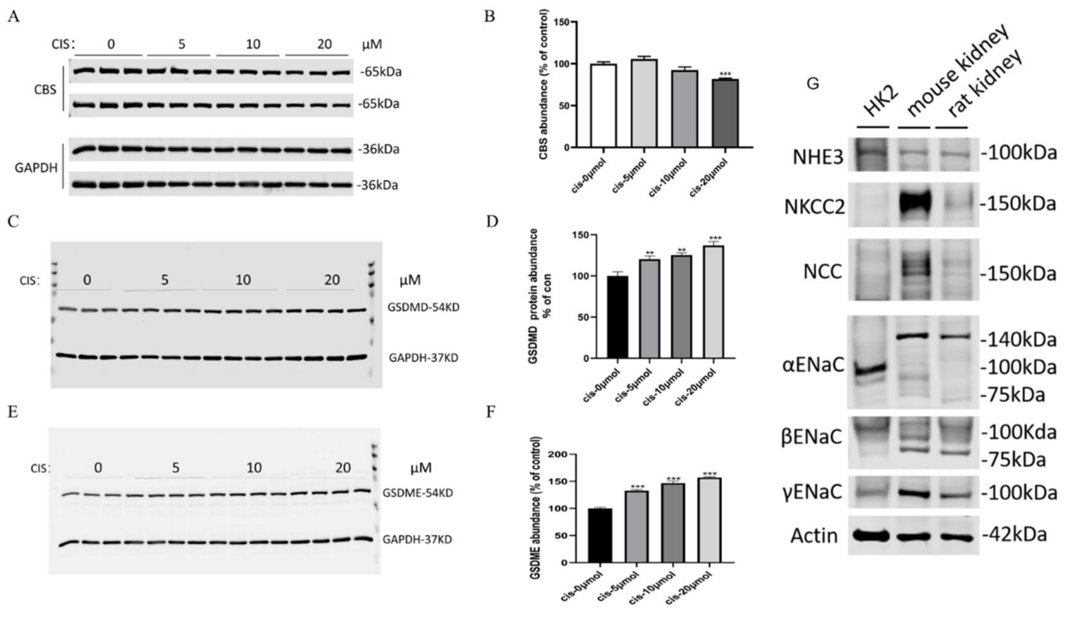

Supplementary Fig.1. Concentration-dependent cytotoxicity of cisplatin and pyroptosis proteins in HK-2 cells.

A: Representative western blot of CBS (n=6). B: Quantitative data on CBS protein levels. C: Representative western blot of GSDMD (n=4). D: Quantitative data on GSDMD protein levels. E: Representative western blot of GSDME (n=4). F: Quantitative data on GSDME protein levels. G: Identification of HK2 cells. Sodium-hydrogen exchanger 3 (NHE3), Sodium-Potassium-Chloride Cotransporter 2 (NKCC2), Sodium-Chloride Cotransporter (NCC), α/β/γ Epithelial Sodium Channel (α/β/γ ENaC). (The results are presented as Mean ± SEM, con vs .cis, *P < 0.05, * *P < 0.01, ***P <0.001. cis vs cis+GYY, # P < 0.05, ##P < 0.01, ###P < 0.001.)

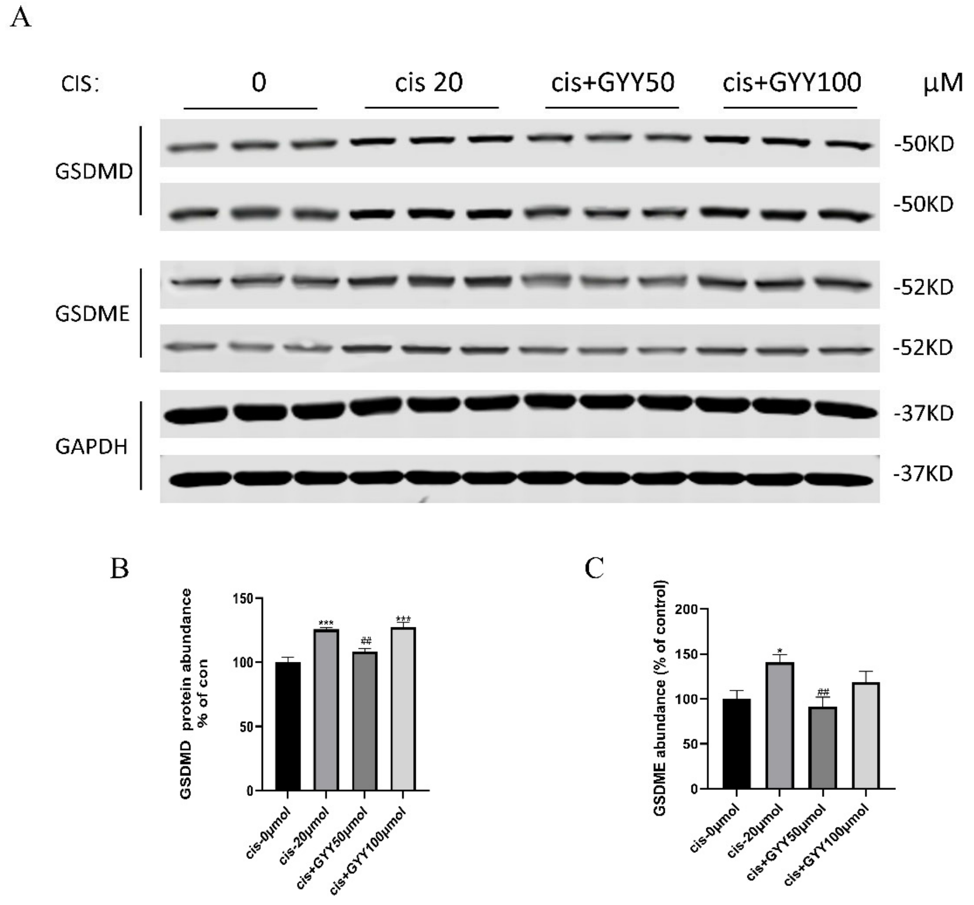

Supplementary Fig.2. Concentration optimization of GYY4137 in HK-2 cells.

A: Representative western blot of GSDMD, GSDME (n=6). B and C: Quantitative data on GSDMD, GSDME protein levels. C: Representative western blot of GSDMD (n=4). Compared with the control group, the expression of pyroptosis proteins increased after cisplatin treatment, while 50μmol of GYY4137 had a protective effect. (The results are presented as Mean ± SEM, con vs .cis, *$P < 0.05$, * *$P < 0.01$, ***$P <0.001$. cis vs cis+GYY, # $P < 0.05$, ##$P < 0.01$, ###$P < 0.001$.)

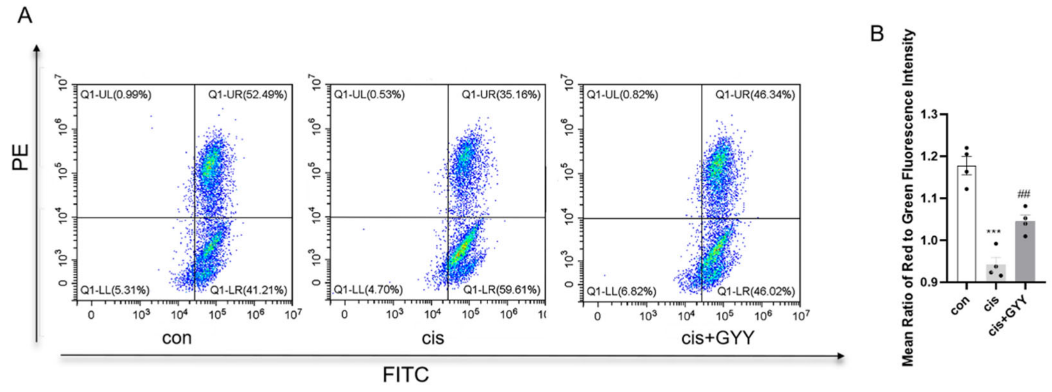

Supplementary Fig.3. Mitochondrial membrane potential of HK-2 cells.

A, B: Mitochondrial membrane potential of cell was detected by JC-1 assay. (n=4). Cisplatin treatment reduced the mitochondrial membrane potential of the cells, which was partially restored by GYY4137 treatment. (The results are presented as Mean ± SEM, con vs .cis, *P < 0.05, * *P < 0.01, ***P <0.001. cis vs cis+GYY, # P < 0.05, ##P < 0.01, ###P < 0.001.)

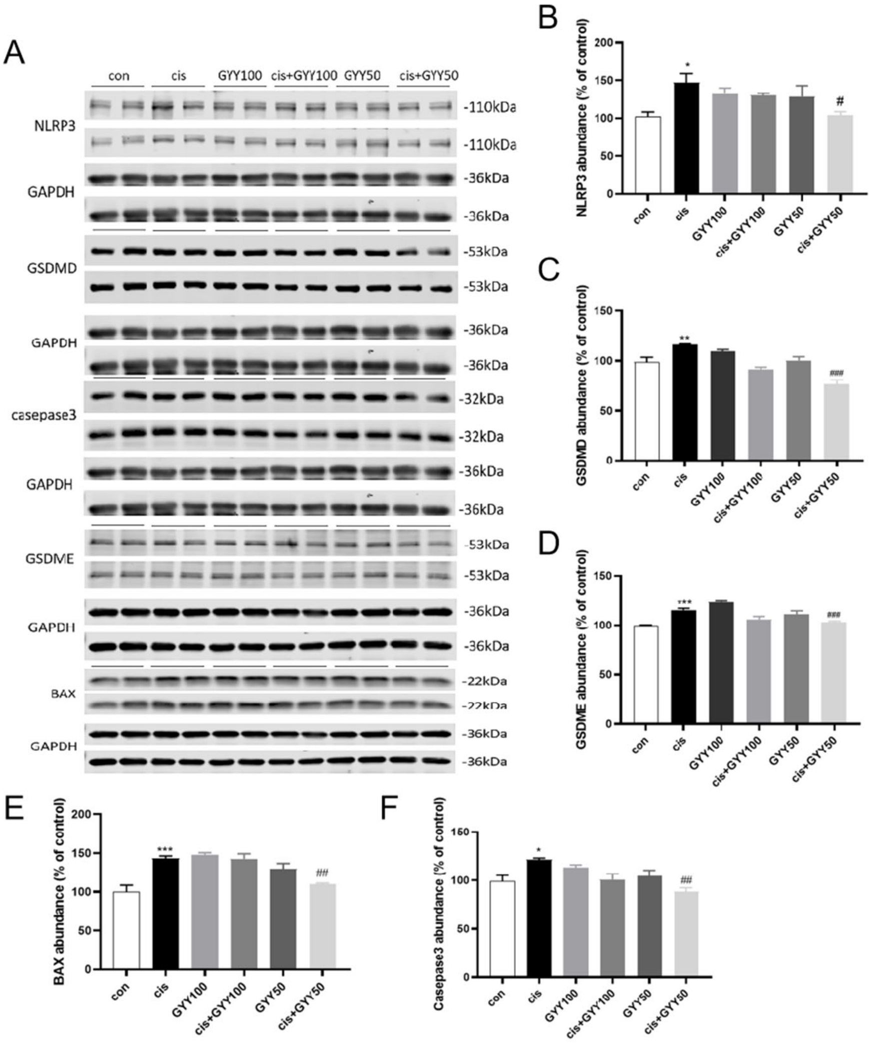

Supplementary Fig.4. GYY4137 modulates cisplatin-induced cell death in mPTCs.

mPTCS were treated with 20μmol cisplatin, 50/100μmol GYY4137, cisplatin-20μmol+50/100 μmol GYY4137. A: Representative western blot of NLRP3/GSDMD, Caspase-3/GSDME, Bax (n=4). B -- F: Quantitative data on NLRP3/GSDMD, Caspase-3/GSDME, and Bax protein levels. Compared with the control group, the expression of apoptosis/pyroptosis proteins increased after cisplatin treatment, while 50μmol of GYY4137 had a protective effect. (The results are presented as Mean ± SEM, con vs .cis, *P < 0.05, * *P < 0.01, ***P <0.001. cis vs cis+GYY, # P < 0.05, ##P < 0.01, ###P < 0.001.)

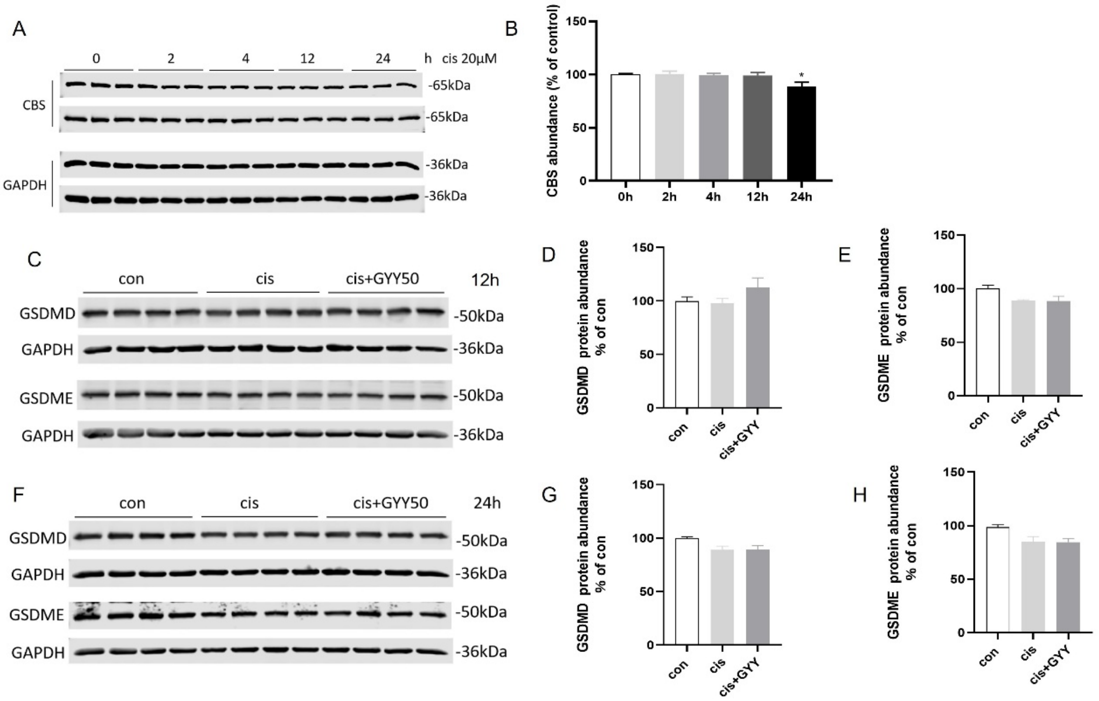

Supplementary Fig.5. Time-course analysis of H_2S-producing enzymes and pyroptosis in HK-2 cells.

A-B: HK-2 cells were treated with 20μmol cisplatin for 0,2,4,12,24 hours. Changes were observed at the 24th hour. Representative western blot/ Quantitative analysis of CBS (n=6). C -- E: HK-2 cells were treated with 20μmol cisplatin with/without 50 μmol GYY4137 for 12 hours. Representative western blot/ Quantitative analysis of GSDMD/GSDME(n=4). F-G: HK-2 cells were treated with 20μmol cisplatin with/without 50 μmol GYY4137 for 24 hours. Representative western blot/ Quantitative analysis of GSDMD/GSDME(n=4). (The results are presented as Mean ± SEM, con vs .cis, *P < 0.05, * *P < 0.01, ***P <0.001. cis vs cis+GYY, # P < 0.05, ##P < 0.01, ###P < 0.001.)

Anti-NLRP3 antibody	Abcam Cat# ab263899, RRID:AB_2889890
Anti-GSDMD antibody	Abcam Cat# ab209845, RRID:AB_2783550
Anti-Bcl-2 antibody	Abcam Cat# ab182858, RRID:AB_2715467
Bcl2 Polyclonal antibody	Proteintech Cat# 26593-1-AP,<br>RRID: AB_2818996
DFNA5/GSDME Polyclonal antibody	Proteintech Cat# 13075-1-AP,<br>RRID:AB_2093053
Caspase 3/P17/P19 Polyclonal antibody	Proteintech Cat# 19677-1-AP,<br>RRID:AB_10733244
BAX Polyclonal antibody	Proteintech Cat# 50599-2-Ig,<br>RRID: AB_2061561
Caspase 1/P20 Polyclonal antibody	Proteintech Cat# 22915-1-AP,<br>RRID: AB_2876874
CBS Polyclonal antibody	Proteintech Cat# 14787-1-AP,<br>RRID: AB_2070970
Rabbit Anti-Human CTH Polyclonal Antibody	Santa Cruz Biotechnology Cat# sc-101924,<br>RRID: AB_2087502
IL18 Rabbit pAb	ABclonal Technology Cat# A20473
IL1β Rabbit mAb	ABclonal Technology Cat# A22257
MCP-1 (E9R7Z) Rabbit mAb	Cell Signaling Technology Cat# 41987
IL-6 (D5W4V) XP Rabbit mAb	Cell Signaling Technology Cat# 12912
GAPDH Polyclonal antibody	Proteintech Cat# 10494-1-AP,<br>RRID: AB_2263076

Supplementary Table 1. RRIDs of antibodies.

Supplementary Table 2. The primers used for qRT-PCR are listed below.

Primer names	species	Gene symbol 5′-3′
Caspae-1 forward	mouse	CTTGGAGACATCCTGTCAGGG
Caspae-1 reverse	mouse	AGTCACAAGACCAGGCATATTCT
NLRP3 forward	mouse	CCTCCAAGACCACTACGG
*NLRP3*reverse	mouse	CGCAGATCACACTCCTCA
GSDMD forward	mouse	TGGGGATGACCTGTTTGT
IL-1β reverse	mouse	CTGCCTGAAGCTCTTGTTG
IL-18 forward	mouse	AGTTGACGGACCCCAAA
IL-18 reverse	mouse	TCTTGTTGATGTGCTGCTGCTG
Caspase-3 forward	mouse	TGTGCTAGAAACGAAAGGG
Caspase-3 reverse	mouse	CCATAAGGAGGCCAGGA
GSDME forward	mouse	TTTATCTGCCACCCTGGA
GSDME reverse	mouse	CCCCGCTCTTATGGTTCT
MCP-1 forward	mouse	ACCTTTTCCACAACCACCT
MCP-1 reverse	mouse	GCATCACAGTCCGAGTCA
IL-6 forward	mouse	AGCCCACCAAGAACGATAG
IL-6 reverse	mouse	GGTTGTCACCAGCATCAGT
Bax forward	mouse	GCCTCGCTCACCATCTG
Bax reverse	mouse	CCCACCCCTCCCAATAA
Bcl-2 forward	mouse	GAGAGCAACCCAATGCC
Bcl-2 reverse	mouse	CGGAGGGTCAGATGGAC
